# How Shall the Regular Medical Profession Be Organized into Ethical Bodies?

**Published:** 1894-09

**Authors:** Daniel Parker

**Affiliations:** Calvert, Texas


For Texas Medical Journal.

### HOW SHALL THE REGULAR MEDICAL PROFESSION BE ORGANIZED INTO ETHICAL BODIES?

BY DANIEL PARKER, M. D., CALVERT, TEXAS.

Read before the Central Texas Medical Association, July 11th, 1894, and published by request.

IF IT were within my province I should be disposed to criticise somewhat the manner in which this question is stated, but as it is not, I shall give myself such latitude as is necessary to present my views on the well worn subject of Ethics.

I understand ethics and morals, or the rules of right-doing, to be practically synonymous terms, and an ethical body of physicians to be an association of physicians who, individually and collectively, practice professional morals, and who are bound together by the talisman of right-doing in all their conduct, both as regards their intercourse with their patrons, the public and one another.

Now, the propriety of such conduct is not in question. It is just as evident that physicians should do right in all their professional relations as it is that "Thou shalt not steal," and it has been wrong to steal ever since there has been anything to be taken. The Decalogue did not create, but simply announced a principle in ethics, as inherent in morals, as gravitation is in physics.

Neither is it a question as to whether the organization of the profession into ethical bodies would be profitable or not. The advantages of organization on such a basis are evident. The question before us is, how it can best be accomplished?

In the first place, "A fountain can not rise higher than its source," neither can you "Make a silk purse out of a sow's ear," which, being applied in this case, means that no society can be better than the individual members of which it is composed, and that you can not make an ethical body out of mean or unprincipled material.

This, like everything else pertaining to morals, is self-evident, and is only mentioned here from the fact that many elementary truths are overlooked, because they are never questioned.

The first thing to do, then, is to elevate the conception of right-doing among the individual members of which the profession is composed, and as far as possible prevent the infusion of improper material through the medium of new recruits.

The present standard to which our conception of medical ethics is required to conform, is the code promulgated by the American Medical Association. Now, while this code is a splendid exposition of the duties of physicians, both to the public and to each other, there is nothing therein commanded or demanded that would not be so maintained by all gentlemen, or which can not be kept in letter and broken in spirit, if the spirit of the gentleman is wanting. For this reason I believe much of the underbrush would be cleared away, a retreat for much unethical conduct destroyed, and our whole conception for right-doing placed on a higher plane if the written code were dropped from our literature, and the only requirement for membership in our society was that the applicant should be a gentleman and a graduate of some school recognized by the American Medical Association.

Christ, as if recognizing the impracticability of tabulating all the meanness that the human heart could practice, and of formulating therefor a "*Thou shalt not*," cuts the whole matter short, and makes each man his own monitor, by announcing a rule so plain, so simple of construction and so evidently correct as to command universal acquiesence: "As ye would that men should do unto you, do ye also unto them likewise."

This Golden Rule represents at the time of its promulgation the moral progress of the fifteen hundred years that had passed since the Decalogue was announced amid the thunders of Sinai. The time was when the human mind was so crudely developed and the conception of right and wrong was so immature, that the unmistakable "Thou shalt" and "Thou shalt not" were necessary enactments; but as moral evolutions displaced the Decalogue with the Golden Rule, so I believe our written code should be displaced by the unwritten law implanted in the heart of every gentleman.

Perhaps some may say that this is letting down the bars too low; that some statutory provisions are necessary to define offenses; that without the basis of a written code, discipline would be impossible, etc.

While this is true of State and municipal organizations, which must be so framed as to govern all classes, and to which all must submit whether they endorse it or no, in organizations like ours, in which membership may be granted or refused without question, and whose cohesive power is represented by its power to mutually benefit and instruct, the standard laid down by the Great Teacher, and of gentlemanly conduct as construed by gentlemen, is, I believe, more effective—the very essence of ethics, and certainly more attractive.

For my part, I would never call upon any society of which I might be a member to discipline a fellow member for any act of a personal character. If I felt that I had been treated discourteously or unprofessionally, I would attend to the matter strictly by myself, and give it such personal attention as, in my opinion, it deserved. When you come to think of it, does it not look a little strange that any one would consult a *code* to learn whether he had been mistreated or not? Or would feel reconciled to misrepresentations or detractions from the fact that it was done by sly inuendos, or a significant shrug of the shoulders, offenses not named in the code?

If I knew of any conduct on' the part of a member calculated to degrade the profession or bring its members into disrepute, I should feel it to be my duty to call attention to the matter, to the end that the individual, and not the profession, might bear the odium. Otherwise, I would never call upon any society to spend its time in matters judicial.

It will no doubt be said, and with some show of reason, too, that there are certain rules the observance of which has been enjoined from time immemorial, that derive their authority not from any moral precept they inculcate, but from the tendency they are presumed to have to elevate the profession and give dignity to its membership; consequently they are peculiar, and applicable to our profession alone, and should be formulated in the shape of a code.

These I would style rules of etiquette, not ethics. To illustrate: It has always been considered derogatory to the character of a physician to advertise; no matter whether it is done in the straight out manner of the tradesman, who announces to the public that he has the best and cheapest goods in the market; or whether a notice is procured in the news column of some local paper to the effect that Mr. A. is rapidly recovering under the skillful treatment of Dr. B.; or that a skilled and delicate operation, calling for a marked degree of dexterity, etc., etc., was performed by Dr. So-and-so, and that it was eminently successful; the object being in both cases to bring somebody into notice, and thereby improve business.

Such conduct has always been considered derogatory to professional character, and has been held to be sufficient to exclude those who practice it from good professional society; just as in social affairs it would be held to be an unpardonable breach of etiquette for any one to write to a person about to give an entertainment of any kind, calling attention to the excellence of his social standing, implying thereby that his presence on the occasion would materially add to its importance. Does any one suppose that the integrity of good society demands a written rule to prevent such practices? Of course not. Neither does the welfare of the profession require any written code to prevent its members from calling attention, through the public print, to their personal or professional excellence. The rules of etiquette, though for the most part unwritten, are well understood and inexorable, and society always finds means to enforce them. So may the profession.

So, in order to elevate the conception of right conduct among the members of the profession, I would dispense with the written code, and place before them the higher standard of just and gentlemanly conduct. I believe the simplicity and beauty of this plan would commend itself to gentlemen everywhere and consequently promote the organization of ethical bodies.

The prevention of the introduction of improper material through the medium of new recruits, is largely a matter of individual effort. It is undoubtedly the duty, and too often neglected, of every physician to discourage, as far as possible, any individual from entering the profession who would be likely to bring discredit on it.

Now, having indicated the ethical standard which alone, I think, should be the basis for the organization of all medical societies, and having drawn a distinction between ethics and etiquette, we are prepared to go a step further. What other attraction is it necessary to offer to induce the gentlemen in the profession to ally themselves with such an organization? I would answer the work must be made profitable and pleasant. An attractive program must be presented. This is a utilitarian age, and any project that does not pay in some way receives slight attention. The men that we want are not going to spend their time and their money and at the same time neglect their business for the sake of meeting, and in a perfunctory way, passing a few resolutions, listening to a stale rehash of some medical topic, or an account of the private differences between doctors A and B.

If proper interest is felt, an attractive program can easily be presented. In the first place those who are placed on the program should never, or at least rarely, disappoint their associates. They should be present to read their papers if possible; but if, as is often the case, they are unable to attend, their paper should be in the Secretary's hands to be read and discussed. It strikes me as rather a humiliating acknowledgment to plead lack of time. Any man competent to practice medicine can write something worth discussing. The consideration of almost any subject allied to medicine may be made interesting if properly managed. The discussion is a part of the program in which all should unite. It is wrong for fifty or a hundred men to sit and hear papers read and have nothing to say about them. It is discouraging to those who have prepared the papers, and deprives those assembled of the most profitable part of the proceedings. All should be prepared to give their views. Young and diffident members should be called out and encouraged to talk. Freedom from restraint should prevail, and thoughts and opinions be interchanged freely. Such a condition would insure success, because it would be profitable. This, at first, must undoubtedly be the work of a few men. In every locality where it would be practicable to organize a medical society, there are a few men who are looked to to take the lead, upon these rests the responsibility of organizing and putting such a society on a successful footing.

The local physicians also of the places where societies meet are charged with special responsibilities. They are not called upon to make the sacrifices in time and money that visitors are, and should not fail to be present and prepared to give the visiting members something profitable to think about when they go home. It is particularly exasperating to the country M. D., who has perhaps ridden twenty miles to take the train for the place of meeting, spent valuable time and hard-earned money, and all for the purpose of rubbing off a little rust by contact with his more favored confrere of the city, and, as it were, take account of stock and size himself up by comparison, to find his city brethren so indifferent as not to attend, or what is about as bad, only make a few pop calls as if to call attention to how extremely busy they are. I am one of those who believe that the more active a physician is in his profession, the more he owes to it, and if he fails to contribute his experience to the common stock, he falls short of his duty, and robs his society of one of the most powerful factors of usefulness and thereby discourages membership.

The social feature of medical gatherings should also receive attention. Man is an animal, and a gregarious one. He loves good cheer and good company, and no one needs or appreciates the relaxatives of a social gathering when the hour is given up to pleasant intercourse more than the care-worn physician. Very often the prospect of a few hours spent in this manner induces him to break away from the bonds of his tread mill life, and is the final inducement to joining some society that offers this attraction. I do not think this feeling is to his discredit.

I have now called attention to the principal means that occur to me as being calculated to assist in organizing the regular profession into ethical bodies, and the purpose of setting them forth more clearly I will recapitulate briefly.

1. I would present to them an organization founded on the highest standard of ethics, viz., on the Golden Rule: and on professional etiquette.

2. I would discourage the consideration or adjustment of all private differences between physicians, trusting every member to guard his own honor, and asking all to guard the honor of the profession, and as far as possible limit discipline to offenses calculated to bring the profession into disrepute.

3. I would offer an attractive program of work, and to this end would make it a point of honor for members to do work assigned them, and would encourage free discussion and free interchange of opinion so as to, as far as possible, make every member feel that he was not only benefited, but was the means of benefiting others.

4. I would especially enjoin it upon the local members of the profession at the places where societies meet, that it is their duty to attend regularly and contribute of their experience freely.

5. I would make the social feature a marked one.

If these suggestions were carried out faithfully I believe it would no longer be a question as to "How the regular profession can be organized into ethical bodies."

